# A Clinical Trial Shows Improvement in Skin Collagen, Hydration, Elasticity, Wrinkles, Scalp, and Hair Condition following 12-Week Oral Intake of a Supplement Containing Hydrolysed Collagen

**DOI:** 10.1155/2024/8752787

**Published:** 2024-07-10

**Authors:** David M. Reilly, Liane Kynaston, Salma Naseem, Eva Proudman, Darcy Laceby

**Affiliations:** Absolute Collagen, 6 Bennetts Hill, Birmingham B2 5ST, UK

## Abstract

**Background:**

Hydrolysed collagen supplements are reported to fight the signs of aging and improve skin appearance, but more authoritative clinical evidence is needed to support efficacy.

**Aim:**

This randomised, double-blind, placebo-controlled study evaluated the efficacy of a supplement containing hydrolysed collagen and vitamin C (Absolute Collagen, AC) on biophysical parameters and visible signs of aging for skin, scalp, and hair, when taken daily or every 48 hours.

**Methods:**

We measured dermal collagen using confocal microscopy and high-resolution ultrasound. Hydration, elasticity, wrinkles, and clinical trichoscopy were measured in parallel to expert visual grading. Efficacy measures were recorded at baseline, week 6, and week 12.

**Results:**

Following 12 weeks daily use of the AC supplement, using confocal microscopy, we observed a significant 44.6% decrease in fragmentation vs. placebo (*p* < 0.01). We also measured a change in the ultrasound LEP (low echogenic pixel) ratio comparing upper and lower dermis (−9.24 vs. −7.83, respectively, *p*=0.05), suggesting collagen improvements occurred more in the upper dermal compartment. After 12 weeks vs. placebo, skin hydration was increased by 13.8% (*p* < 0.01), R2 elasticity index was increased by 22.7% (*p*<0.01), and Rz profilometry index was decreased by 19.6% (*p* < 0.01). Trichoscopy showed an average 11.0% improvement in scalp scaling and a 27.6% increase in the total number of hairs counted vs. placebo (*p*=n.s.). This was associated with a 31.9% increase in clinical grading score for hair healthy appearance (*p* < 0.01).

**Conclusion:**

The AC supplement has shown clinical benefits for skin, scalp, and hair, when used either daily or every 48 hours, over a 12-week period.

## 1. Introduction

Collagen is the most abundant protein in the skin, but its importance runs much deeper than this simple fact. As a protein, collagen is uniquely made for strength (structural rigidity imparted by proline), stability (construction of triple helices), and resilience imparted by extensive crosslinking [1, 2]. The formation of dense fibrous networks is pivotal for production of an extracellular matrix scaffold with high tensile strength for the skin [3].

Collagen, by way of interaction with elastin and glycosaminoglycan networks, contributes to elasticity, firmness, and skin hydration. Collagen production declines with age and is driven by intrinsic factors (e.g., genetics, hormonal fluxes, and metabolic dysfunction) and extrinsic factors (e.g., UV-radiation, pollution, and smoking) [4]. In the aging skin, the ability to replenish collagen naturally decreases by about 1.0% per year [5]. This decrease in collagen has been linked to the appearance of fine lines and deeper wrinkles.

Stimulation of collagen production and/or inhibition of collagen degradation can be achieved via the use of oral supplements, designed to deliver skin benefits and often referred to as “nutricosmetics.” Supplements containing hydrolysed collagen (HC) are reported to fight the signs of the aging process, contributing to a youthful and healthy appearance. When consumed, HC is further hydrolysed to amino acids, dipeptides, and tripeptides. Due to the very high content of hydroxyproline, proline, and glycine in collagen, the resultant peptide species are unique and will not be typically found in other protein sources (e.g., milk and plant-based or fermentation-based vegan equivalents) [6]. These peptides appear in the bloodstream and skin following ingestion and are ligands proven to stimulate fibroblasts to synthesise new collagen proteins [7, 8].

The HC should be combined with vitamin C (l-ascorbic acid) which, in addition to its role as an antioxidant, can support the synthesis of the collagen triple-helix protein and the assembly of collagen into fibres. The importance of l-ascorbic acid is reliant on its use as a cofactor in hydroxylation of proline residues in procollagen (which stabilises the triple-helix structure) and lysine residues (which are used to cross-link fibres) [9, 10]. As an antioxidant, l-ascorbic acid can interact with a range of free-radical species to facilitate their neutralisation. This is a key mechanism by which the skin is protected from free-radical damage due to UV-radiation and inflammaging [11].

There have been several reviews of published randomised clinical trials (RCTs) assessing the efficacy of HC-based supplements. A review by Choi et al. carried out a systematic review of 11 studies, with a total of 805 subjects using oral collagen supplementation, and showed significant benefits on skin elasticity, hydration, and collagen density [12]. Al-Atif summarised the findings from 12 RCTs that demonstrated improved skin elasticity, turgor, hydration, and reduced skin wrinkling and roughness [13]. Pu et al. reported on 26 RCTs assessing the efficacy of the product tested in a total of 1721 subjects and reported benefits of skin hydration and elasticity [14]. The variability of doses, age range, number of weeks tested, and skin measures does little to support the robustness of the clinical reports. However, there is a general pattern suggesting that HC is efficacious as an antiaging treatment for women who use the supplement for up to 12 weeks [15, 16]. The conclusion of these systematic reviews and meta-analysis is that HC supplementation can improve skin hydration, elasticity, and wrinkles.

Whilst the clinical benefits of HC for the skin are well-documented, many of the trials are lacking in a detailed analysis of skin structures such as collagen and therefore do not support the link between the proposed mechanism of collagen production and the putative skin benefits. There is also a lack of validated trichological assessments of the scalp and hair, which ideally should be carried out on the same cohort, in the same clinical trial.

The use of HC supplements for consumers who wish to benefit from improved skin and hair quality, coupled with the proposed ability to fight the signs of aging, has increased rapidly in the last several years. There is a need for larger trials with more technical and clinical measures to support this antiaging approach.

We have conducted a clinical trial showing the skin, scalp, and hair benefits of the AC supplement, when used daily, or every 48 hours, and tested against a placebo. To the best of our knowledge, this is the first report of the efficacy of collagen peptides to improve the skin, scalp, and hair, in the same cohort. For the first time, we have used high-resolution ultrasound images to show that there are differential effects following 12 weeks use of the supplement, with increased collagen content in the upper (papillary) dermis compared to the lower (reticular) dermis. Finally, we report the dose regimen is critical to ensure maximal clinical efficacy, with daily use being far superior to use every 48 hours.

## 2. Methods

### 2.1. Study Design

This study was a randomised, placebo-controlled, double-blind design following the principles of Good Clinical Practice. The protocol was approved by an independent Ethics Committee (signed by the chairman of the East Anglia Ethics Committee on 09.01.2023). A written declaration of informed consent was received from all participants. The study conformed to the requirements of the 1964 Declaration of Helsinki.

### 2.2. Subjects

**Figure 1:**
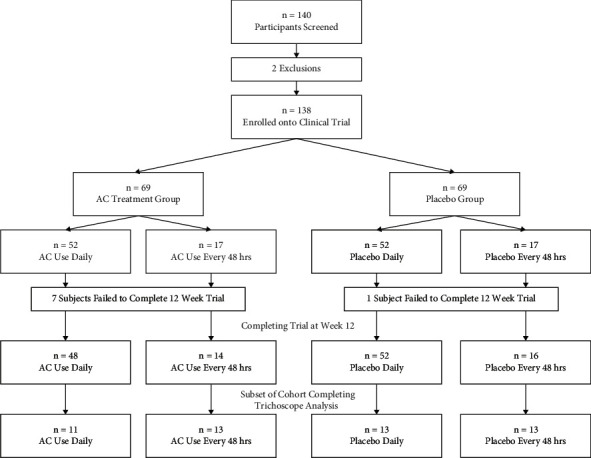
Clinical flowchart. Trial design, including subject allocation and participation over 12 weeks.

A total of 140 male and female participants (aged 40–60 years) were enrolled in this study, with 130 participants completing the 12-week trial (Figure 1). The inclusion criteria included visible signs of aging, requiring a minimum Glogau Wrinkle Score of 2 and a hyperpigmentation score of 4. Fitzpatrick skin types I–VI were recruited, and the split between genders was 90% female and 10% male. Menopausal status was recorded for the female cohort. Exclusion criteria included current skin disease on the face or scalp, allergy or intolerance to test product, aesthetic skin surgery (including Botox), and medications such as anti-inflammatory drugs applied directly to the test area (face/scalp).

### 2.3. Test Products

AC is a skincare supplement containing 8000 mg of hydrolysed collagen, sourced from a sustainable marine supply. The supplement also contains 60 mg l-ascorbic acid (75% NRV). A placebo product was manufactured to match the organoleptic properties of the AC supplement, including taste, texture, colour, and viscosity.

For blinding of the clinical trial, both products were presented in a plain white sachet, with only an assigned product code to identify each batch. The sachets were identical in appearance and labelled according to the ICH-GCP requirements. Subjects were instructed to take 1 sachet per day, or 1 sachet every 48 hours, preferably in the morning, on an empty stomach. Personnel who were not involved in any other aspect of the study were tasked to label the investigational product. The investigators, statistician, other site personnel, and participants were blinded to the product or placebo. The unblinding of the clinical trial only occurred after the data were analysed and reported using coded identifiers (participant numbers) and coded parallel arm identifiers (groups 1–4, representing AC or placebo taken daily and AC or placebo taken every 48 hours). A home use diary was used for compliance checks.

### 2.4. Measurement Conditions

Subjects equilibrated in a controlled environment (at a temperature of 20°C and at a relative humidity of 25–40%) for at least 15 minutes prior to any assessments being performed. Measurements were taken on the infraorbital region (below the eye socket) for the corneometer and cutometer and on the lateral canthal lines (crow's feet area) for the confocal microscopy, ultrasound, and profilometry.

### 2.5. Confocal Microscopy

**Figure 2:**
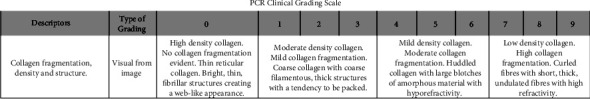
Grading of confocal images used to quantify collagen density, structure, and fragmentation.

The collagen structure, specifically the quality, density, and fragmentation in the papillary and superficial dermis, was visualised using a confocal laser scanning microscope (VivaScope® 1500; GmbH, Munich, Germany). The 3D stereoscopic images allow the cellular microstructure to be visualised via optical cross sections with a horizontal resolution of up to 1.25 *μ*m and vertical resolution of 3–5 *μ*m. This can be used to target a depth of the superficial dermis of ∼200 *μ*m. Approximately 40 individual images were acquired at 5 *μ*m interval scanning steps to create a stereoscopic stack. Only the images targeting the collagen network in the papillary and superficial dermis of the lateral canthus (crow's feet area) of the face were analysed. Images taken at baseline were compared to those taken at week 6 and week 12. The images for each were graded for collagen structure by an expert grader, based on evaluation of a blinded set of confocal images. Collagen density, structure, and fragmentation are based on a composite scoring system using a Visual Analog Scale (VAS). As can be seen in Figure 2, a score of 0 represents high density collagen, clear evidence of collagen structure, and with no visible fragmentation. A score of 9 represents low density collagen, lack of structured collagen fibres, and clearly visible fragmentation.

### 2.6. Ultrasound

The ultrasonographic evaluation was performed at baseline, week 6, and week 12, at the lateral canthus area of the right side of the face (crow's feet area). The Dermascan-C device (Cortex Technologies, Aalborg, Denmark) is equipped with a 20 MHz transducer that allows sectional skin images up to a depth of 2.5 cm. The transducer was applied perpendicularly on the skin, and the gain curve was adjusted at a value of 20 dB and a velocity of propagated sound wave of 1580 m/s. The obtained images were processed with image analysis software provided by Dermavision, Cortex Technology. The Dermavision software used pixel amplitude corresponding to a numerical scale set between 0 and 255. The 0–30 interval corresponds to low echogenic pixels (LEPs, collagen degeneration), the 50–150 interval to medium echogenic pixels (MEPs, collagen and elastin), and the 200–255 interval to high echogenic pixels (HEPs, collagen and elastin). The number of LEP was determined separately in the upper (LEP_u_) and lower (LEP_l_) dermis by dividing the dermal image into two parts of equal thickness. The ratio of LEP_u_/LEP_i_ was calculated and allows an assessment of the integrity of the collagen in distinct dermal compartments.

### 2.7. Skin Hydration

Skin hydration measurements were performed using the Corneometer® CM825 (Courage and Khazaka, Germany) at baseline, week 6, and week 12. This instrument relies on the dielectric constant of water. Any change in the dielectric constant due to skin moisture variations will alter the capacitance of the instrument. These variations are detected electronically and are converted into a value represented as arbitrary units.

### 2.8. Elasticity

The viscoelastic properties of the skin (elasticity and firmness) were performed using the Cutometer® MPA 580 (Courage and Khazaka, Germany) at baseline, week 6, and week 12. The measuring principle is based on the suction method, in which negative pressure is created in the device and the skin is drawn into the aperture of the probe. The measurements for deformation, passive stretch, and recoil were recorded. For each measurement, a constant suction of 400 mbar was applied for five seconds, followed by a relaxation time of 3 seconds, measured in triplicate. Elasticity is defined as the skin resistance to mechanical suction force (Ua) vs. its ability to recover after release (Uf) where R2 = Ua/Uf.

### 2.9. Profilometric Analysis

A skin surface replica was taken of the crow's feet area of the left side of the face at baseline, week 6, and week 12. A negative replica of the wrinkles on the cutaneous surface of the crow's feet area was obtained using Silflo® (JS Davis, Hart) material. Profilometric analysis was carried out using the OPTIMAS v6.5 in combination with StatSoft STATISTICA 7. Luminance was measured along a set of 10 equal length parallel lines (passes) running across the replica parallel to the lighting direction. The variations in luminance were treated as indicative of the roughness and analysed by traditional surface roughness statistics, with Rz reporting the average maximum difference in the luminance value for five equal length segments in each of the 10 lines traversing the sample. The ISO definition of Rz (ten-point height of irregularities) is the average value of the absolute values of the heights of five highest profile peaks and the depths of five deepest valleys within the evaluation length. A reduction in the Rz index is indicative of a reduction in fine lines and wrinkles at the crow's feet area.

### 2.10. Skin Visual Grading

The Glogau Scale is a validated dermatological tool evaluating skin photoaging, including wrinkles, pigmentation, keratosis, and other skin imperfections. Expert clinical staff applied this 4-point VAS to participants viewed under standardized lighting conditions. Grade 1 corresponds to mild damage to the skin, whereas Grade 4 corresponds to severe photoaging. Expert visual assessors also graded subjects according to evenness of skin tone. A 9-point VAS (where 0 = even skin tone and 9 = very uneven skin tone) was used to follow the putative effects of the AC supplement to improve skin characteristics when compared to a placebo.

### 2.11. Trichoscopy

The DinoLite Pro Polarising Trichoscope (Dinolite, the Netherlands) was used to capture images of the hair and scalp. For each subject, 3 areas of the scalp were measured, specifically the front, mid, and back of the scalp along a central parting to allow capture of high-resolution images. The images were loaded into analytical software TrichoScience Pro V 1.7 for measurement and data recording.

The centre section of the scalp was chosen as the measurement area, with measures taken at 4 cm intervals from the hairline to the crown, to assess the scalp condition and the total hair count. This was carried out at the start of the trial and repeated at the end of the study at 12 weeks. The key measurements for the scalp were inflammation and scaling, and for hair, we measured the average total hairs per unit area.

Scalp inflammation was measured on a 5-point VAS, where a score of 1 represents inflammation measured at all 3 test sites and a score of 5 represents normal, healthy, uninflamed scalp. Scalp scaling was measured on a 5-point VAS, where a score of 1 represents scaling that covers more than 30% of all 3 test sites and a score of 5 represents normal, healthy scalp with no visible flakes. The TrichoScience Pro V1.7 is set to automatically count the total hairs per unit area, and this assessment is carried out for each of the 3 test sites and then averaged. Data are presented as number of hairs per unit area (cm^2^).

### 2.12. Hair Visual Grading

Expert visual grading of hair healthy appearance was conducted at baseline, week 6, and week 12. Assessments were made with the aid of a 60-Watt pearl bulb, with distance maintained at approximately 30 cm from the test site. Hair healthy appearance was measured on a 5-point VAS, where a score of 0 represents maximum healthy appearance (rated according to the parameters thick, voluminous, soft, and shiny) and a score of 4 represents poor hair appearance.

### 2.13. Self-Perception Questionnaires

The subjects completed self-perception questionnaires (SPQ) at baseline, week 6, and week 12. The SPQ documented their perception of attributes for the skin, scalp, and hair, after use of the test article or placebo, daily or every 48 hours. A total of 35 questions were presented at week 6 and week 12, in addition to allow the subject to present their own feedback about the product use and efficacy. Subjects rated the test article, skin and hair improvements, and intensity of clinical efficacy by ticking a box labelled strongly agree, agree, neither agree nor disagree, and disagree or strongly disagree.

### 2.14. Data and Statistical Analysis

The sample size calculation was based on the primary objective of the comparison of 12-week skin confocal fragmentation changes between the treatment and placebo groups. With an estimated 80% power, 5% significance, and 10% attrition, 100 participants were required (*n* = 50 per group). This calculation assumed variability of confocal density and fragmentation data based on an earlier clinical trial [17].

For profilometry Rz, cutometer R2, corneometer, and ultrasound analysis, we used descriptive statistics (mean and standard deviation) and change from baseline (*t*-test, mean percent change from baseline). For statistical comparison considering treatment vs. placebo, we applied a linear regression analysis (ordinary least squares) model. For the VAS measures including VivaScope 1500, hyperpigmentation, Glogau Scale of wrinkling, photodamage, and hair attributes, we used as above except for the change from baseline (Wilcoxon signed-rank test). For the SPQ responses, percentage of the top 2 positive responses (strongly agree + agree) was reported. The frequency of “top 2 box” responses was determined for each question. The significance of frequency responses was determined using a chi-squared test with *a priori* 50/50 distribution assumption.

## 3. Results

### 3.1. Study Cohorts, Adverse Events, and Protocol Deviations

A total of 140 volunteers were screened and 138 eligible participants were enrolled, randomised, and split into 4 parallel cohort test groups, comprising treatment vs. placebo, for both use daily or use every 48 hours (Figure 1). Two subjects failed to meet inclusion/exclusion criteria, and a further 8 subjects failed to complete the trial. Thus, the final count was 48 subjects taking AC supplement daily vs. 52 taking placebo daily and 14 subjects vs. 16 subjects taking the AC supplement or placebo every 48 hours, respectively. A subset of each group (*n* = 11 taking the AC supplement daily vs. *n* = 13 for the other groups) was assigned for clinical trichoscopy. No adverse events were recorded, as expected for a dietary supplement. Two protocol deviations were assessed. A target representation of 20% male participants was not met, as only 10% of male participants who applied and met inclusion criteria were recruited onto the study. One subject was recruited onto the clinical but later an error was found in which the participant was outside the age range for the clinical and was therefore removed from further analysis. The assessment concluded that these protocol deviations did not significantly affect the study.

### 3.2. The AC Supplement Improves Collagen Density and Reduces Fragmentation

**Figure 3:**
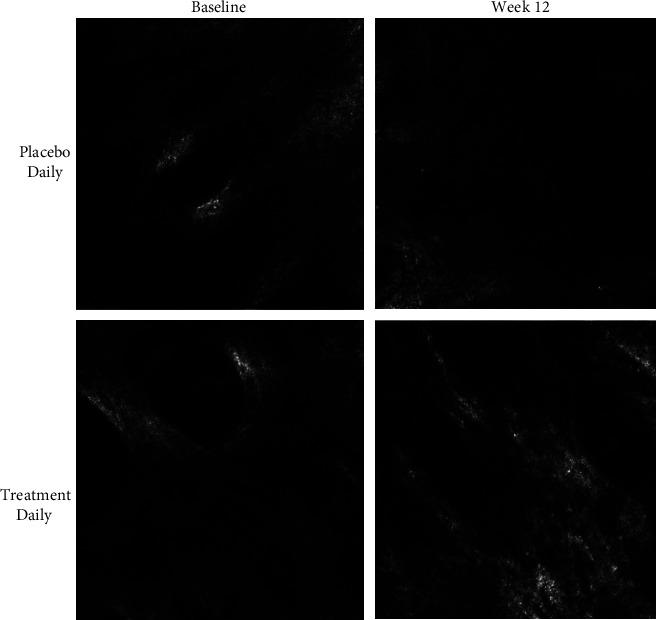
Effect of supplement on the collagen density, structure, and fragmentation. Representative images from 1 subject in the treatment group and the placebo group are shown. Confocal images show low collagen density, poor structural integrity of fibres and a visible fragmentation at baseline for both groups. At week 12 there is an increase in collagen density, increased presence of fibrillar structures and a decrease in the fragmentation pattern for subjects using the AC supplement daily.

Expert visual grading of confocal images generated from the VivaScope showed a trend of decreasing collagen fragmentation, corresponding to an improvement in collagen density and structural integrity (Figure 3). As shown in Table 1, following daily use of the AC supplement for 6 weeks, the fragmentation VAS grade had decreased 25.3% compared to the placebo, which showed only minor changes (*p* < 0.01). By week 12, the fragmentation VAS grade had decreased 44.6% vs. placebo (*p* < 0.01). Thus, the collagen fibres were increased in density and less fragmented after daily use of AC vs. placebo.

### 3.3. Ultrasound Shows That AC Supplement Improves Collagen Content

By week 12, with daily use of the AC supplement, the HEP signal had increased 231% from baseline (*p* < 0.01) compared to the placebo which increased 176% from baseline (*p* < 0.01). The difference between treatment and placebo was not statistically significant. By week 6, the LEP_u_/LEP_i_ ratio had decreased 16.3% from baseline, compared to a 9.5% decrease for the placebo group (*p* < 0.01). By week 12, the LEP_u_/LEP_i_ ratio had decreased 9.3% from baseline compared to a 7.8% decrease for the placebo (*p*=0.05).

### 3.4. Use of AC Supplement Improves Hydration, Elasticity, and Wrinkles

**Figure 4:**
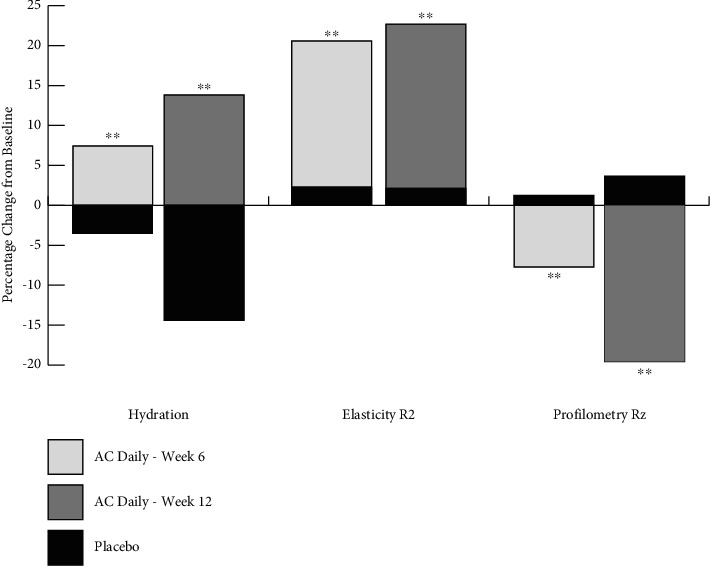
Summary of skin parameters for hydration, elasticity, and wrinkles. For comparative purposes, data for hydration, R2 and Rz were normalised by expressing as percent change from baseline. Each parameter was expressed relative to placebo. With daily use of the AC supplement, hydration and elasticity showed an increase (i.e., an improvement in skin quality) both at week 6 and week 12 compared to placebo (*p* < 0.01). Wrinkle size, Rz, was reduced (i.e., less visible in size) both at week 6 and week 12 compared to placebo (*p* < 0.01) (^*∗∗*^denotes *p* < 0.01).

As summarised in Table 1 and Figure 4, using the AC supplement daily, at week 6, the hydration had significantly increased 7.5% vs. placebo (*p* < 0.01). By week 12, a further increase in hydration to 13.8% was measured compared to placebo (which decreased 14.4%, *p*<0.01). Elasticity was measured as the skin resistance to the mechanical suction force (Ua) versus its ability to recover after release (Uf), where R2 = Ua/Uf. By week 6, for subjects taking the AC supplement daily, R2 significantly increased 20.6% vs. placebo (*p* < 0.01). By week 12, the R2 had further increased 22.7% vs. placebo (*p* < 0.01). For measurement of fine lines and wrinkles, the Rz parameter of the profilometry method was used, as this is the accepted clinical standard by which the impact of HC supplement use is measured. By week 6, for subjects taking the AC supplement, the Rz index had decreased 7.7% vs. placebo (*p* < 0.01). By week 12, the Rz index had decreased 19.7% vs. placebo (*p* < 0.01).

### 3.5. Expert Visual Grading and Self-Perception of AC Efficacy for Skin Appearance

As can be seen in Table 1, after 12 weeks of daily use of AC supplement, the Glogau Score had improved by 8.54% compared to placebo (*p* < 0.01). VAS score for expert grading of skin tone evenness showed a 31.9% improvement in skin tone vs. placebo (*p* < 0.01). By week 12, 87.5% of subjects reported that their skin felt more hydrated (vs. 63.4% in the placebo group), 72.9% of subjects reported their skin felt firmer (vs. 50% in the placebo group), 62.5% reported that their wrinkles appeared reduced (vs. 36.5% in the placebo group), and 62.5% of subject reported their skin had more elasticity and felt stronger from within (vs. 28.8% in the placebo group). SPQ responses were statistically significant (*p* < 0.05) except for the self-perception of wrinkle reduction.

### 3.6. Clinical Trichoscopy, Visual Grading, and SPQ Show AC Use Improves Scalp and Hair Condition

A trichoscope camera system was used to magnify scalp surface features and hair follicles up to 60-fold. The scalp was assessed for inflammation, flaking, plugging, and scaling. When using the AC supplement daily for 12 weeks, we measured an average increase of 11 ± 29% improvement in VAS scalp scaling vs placebo (no change). This corresponded to an average increase of 33.4 ± 26.2 total hairs counted per unit area (representing a 27.6% increase vs. placebo, *p* = n.s.). A detailed clinical analysis of scalp sebum, follicular plugging, and inflammation, in parallel to total hairs per follicular unit and mean hair diameter, shows improvements when using the AC supplement daily (manuscript in preparation for trichology publication).

By expert visual grading, hair healthy appearance was assessed on a 5-point scale. VAS score for hair healthy appearance improved 31.9% for subjects taking the AC supplement daily vs. 9.4% of subjects who took placebo daily (*p* < 0.01). By week 12, SPQ reported that 66.7% of subjects felt their hair thicker vs. 44.2% in the placebo group (*p* = 0.02), whereas 77.1% of subjects reported that their hair felt smoother vs. 55.8% in the placebo group (*p* < 0.01).

### 3.7. AC Use Daily Shows Superior Efficacy Compared to Use Every 48 hours

**Figure 5:**
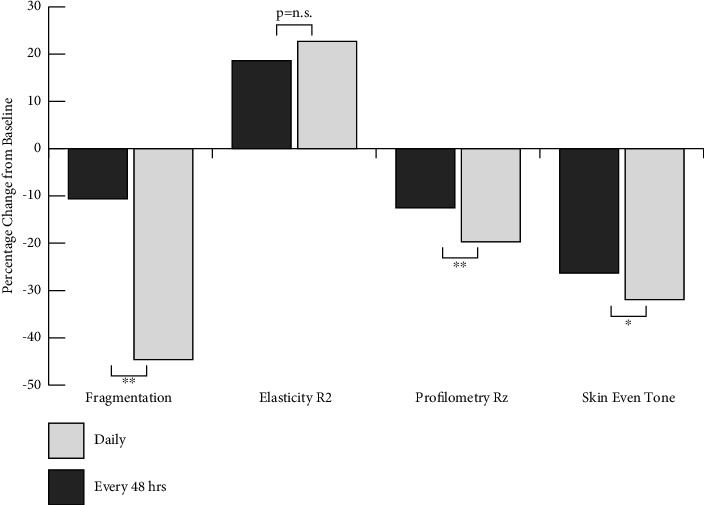
Summary of AC use daily vs. use every 48 hours. For comparative purposes, data for collagen density and fragmentation, the elasticity R2 index, profilometry Rz index (wrinkles), and expert visual grading of skin even tone (pigmentation distribution) are expressed as percent change from baseline. Each parameter was expressed as daily use of the supplement or use every 48 hours (^∗^*p* < 0.05; ^∗∗^*p* < 0.01).

As can be seen in Table 2 and Figure 5, for subjects consuming the AC supplement daily, by week 12, the fragmentation VAS grade had decreased 44.6% compared to subjects taking the supplement on every second day, in which case fragmentation grades had decreased 10.6%. Daily use of the AC supplement showed significantly improved collagen density, integrity, and a reduction of fragmentation compared to use every 48 hours (*p* < 0.01); that is, the difference between the 2 treatment regimens was statistically significant.

For subjects taking the AC supplement daily, by week 12, the elasticity index, R2, had increased 22.7% compared to subjects taking the supplement on every second day, in which the R2 index had increased 18.6%. The difference between the 2 treatment regimens was not statistically significant. By week 12, for subjects taking the AC supplement daily, the Rz index had decreased 19.7% compared to the group taking the supplement on every second day, in which the Rz index had decreased 12.5%, with the difference being statistically significant (*p* < 0.01). Expert grading VAS score for skin tone evenness showed a 31.9% improvement at week 12 compared to the group taking the supplement on every second day, which improved 26.3%, with the difference being statistically significant (*p*=0.03).

### 3.8. Exploratory Postsupplementation Phase

After the 12-week clinical trial ended, subjects discontinued the use of the AC supplement. A subset of 32 participants returned after another 8 weeks of additional skin measures (hydration, elasticity, and wrinkles) and self-perception feedback. Furthermore, the data for week 12 are different for the full clinical study because we only compare the scores for the 32 participants of this exploratory phase of the RCT (as opposed to the full clinical study which tested 48 participants).

After taking AC supplement for 12 weeks, hydration had increased by 11%, but when the participants stopped taking AC at week 20, it had decreased by 4%. Elasticity R2 index had increased by 53% at week 12, but at week 20, the R2 index dropped to only 31%. Wrinkles had decreased by 20% at week 12, whereas at week 20, the Rz index had dropped to 8%. This indicates that when subjects cease using the supplement, then there is a measurable loss of skin benefits. This exploratory part of the study will be used for a statistical power analysis to estimate the minimum sample size required for a follow-up clinical trial.

## 4. Discussion

Photodamage is characterised by many attributes including leathery texture, thin, flattened and dry features, fine lines and wrinkle formation, poor elastic recoil, fragility of the skin, and altered pigmentation due to haemoglobin and melanin [18]. For over a decade, a key hypothesis proposes that many of these problems are putatively related to loss of collagen, which can be reversed by the ingestion of hydrolysed collagen supplements [19].

In this randomised, double-blind, placebo-controlled clinical trial, we have provided evidence linking the use of hydrolysed collagen supplements, with increased collagen content and quality in the skin. We demonstrated that this increased collagen in the skin is associated with improved appearance indices such as hydration, elasticity, and wrinkles. The study was run during Q1, 2023 to effectively minimise variability due to seasonal changes, especially increased UV-radiation during summer and anticipated damage caused by UV-radiation to both dermal (collagen fragmentation and wrinkle formation) and epidermal (hydration and pigmentation) features.

Collagen is a uniquely stable and long-lived protein with a half-life reported to be approximately 15 years [20, 21]. Over that duration of time, it can be influenced by extrinsic and intrinsic factors, including UV-radiation, glycation, hormonal flux, oxidative stress linked to free-radical attack, mechanical shear stress, and physical fatigue. In addition to direct damage, the production of matrix metalloproteinase (MMP) enzymes, in particular collagenase, can cleave the collagen triple helix and make the fibre accessible to degradation enzymes and cellular recycling [22]. The collagenases are critical to carry out the first degradation step, in which the fibres are cleaved into characteristic 1/4 and 3/4 fragments [11, 23]. The resultant fragments can be visualised, quantified, and represent collagen damage in the dermis.

The collagen fibres *in situ* can be described as hyporeflective (where it is not possible to identify single fibres), hypereflective (in which a well-defined, fibrous collagen can be observed), thin reticulated collagen, coarse collagen, huddle collagen, and curled bright structures [24]. According to Bhardwaj et al., short fragmented collagen and a huddled arrangement are typically seen in cases of damaged and poorly oriented collagen in aged or photodamaged skin [25].

Both the amount and quality of the collagen network changes over time, with state-of-the-art confocal laser scanning microscopy (CLSM) being used to visualise and quantify such changes. We have presented images of the dermis in the crow's feet area of the face at baseline and 12 weeks after the oral intake of AC supplement vs. placebo. At baseline, there is low collagen density observed and a lack of structural fibres which usually represent collagen-based ECM. This corresponds to visible evidence of fragmented collagen which has been reported to underline loss of skin elasticity and appearance of facial wrinkles. Following daily use of the AC supplement, an increase in collagen density and structural integrity can be observed in parallel to a decrease in fragmentation. This improvement in dermal collagen content was not observed in the placebo group. This is in agreement with week 12 results presented by Asserin et al. [17], albeit we have shown the effect occurring as early as week 6, with progressive improvements over the 12-week period. We have also shown, for the first time, that this benefit is dependent on the dosing regimen.

In addition to fragmentation, it is important to consider the density and quality of collagen in the dermis, paying particular attention to the stratification of collagen in relation to papillary and reticular fibroblast populations [26, 27]. Papillary fibroblasts in the upper dermis have been reported to secrete distinctly discernible thin, poorly organized bundled collagen fibres, compared to the reticular fibroblasts which secrete thick, well-organized collagen bundles. There is also a distinct pattern of the papillary ECM exhibiting a higher ratio of collagen type III to type I, a pattern often observed in younger skin and also in wound healing responses [11, 28].

The characteristic organized, parallel-oriented, and bundled collagen fibrils are one of the key echogenic structures of the dermis. The ultrasonographic markers included % pixel area (corresponding to collagen density), LEP, MEP, HEP, and the ratio of LEP_u_/LEP_i_. The LEP indicator quantifies the degree of cutaneous hydration, inflammatory processes, solar elastosis, and collagen degeneration. The MEP and HEP indices quantify the structures of collagen and elastin fibres and microfibrils. In general, an increase in collagen density, MEP, and HEP was observed (all which correspond to increased collagen in the dermis), although only the HEP showed an improvement compared to placebo. The variability in echogenic markers and strong placebo effect we hypothesise is due to the superimposition of innate collagen turnover mechanisms in combination with effects of the supplement.

The increase in HEP pixel data is indicative of increased collagen content and quality in the dermis. When considered in the context of a decrease in the LEP_u_/LEP_i_ ratio, we surmise that this is evidence of improvements in the papillary and upper dermis. This is in agreement with previous publications on changes on the LEP_u_/LEP_i_ ratio in the aged skin and following treatment with antiaging topical treatments (such as the topical flavonoid and *ε*-viniferin) but needs to be independently confirmed in the case of daily use of HC supplements [29–31].

Many of the published antiaging, hydrolysed collagen clinical trials, do not add vitamin C to the product being tested. We regard the inclusion of vitamin C as a key factor driving efficacy of these supplements. The role of vitamin C (l-ascorbic acid) as a cofactor required in the synthesis of collagen and the ECM is well documented, but the importance of this vitamin as an antioxidant is often overlooked. Free radical damage to the skin is triggered by several mechanisms including UV-radiation, mitochondrial oxidative respiration, glycation, and lipid oxidation. Ascorbic acid neutralises a diverse number of free-radical species to facilitate their detoxification [32]. The consequent generation of a stable ascorbyl free radical yields a far less reactive species which effectively minimises free-radical damage and protects the long-lived collagen fibres in the skin [33].

Linking changes in dermal collagen content to phenotypic and visible skin attributes is important to the overall acceptance and credibility of the use of hydrolysed collagen-based supplements. Studies using profilometry show the architecture of the uppermost layers of the skin, either as microrelief (including fines lines) or larger structures such as wrinkles. The skin microrelief changes with age independently of wrinkle formation, and thus any model of profilometric analysis of the skin needs to account for these independent characteristics. Fine lines and wrinkles were significantly decreased at both timepoints, and this improvement was not observed in the placebo group.

Skin hydration and elasticity were significantly increased after 6 weeks and 12 weeks use of the AC supplement, in direct contrast to the placebo which showed negligible change from baseline, or even a deterioration of skin parameters at both timepoints. The effects of supplement on elasticity are likely due to changes in collagen content in addition to changes in elastin and glycosaminoglycan content of the dermis. The decrease in hydration observed in the placebo group is likely due to the fact that the clinical trial was run during the winter season, where many people experience some degree of dry skin/winter xerosis, associated with low temperature and low humidity.

The use of supplements as a support to a healthy and varied diet is an issue of popular debate. A key question arises as to the frequency of use of the supplement and specifically whether daily use of the will yield more, or less, skin benefits compared to less frequent use (e.g., every 48 hours). We tested this hypothesis in a parallel cohort to the main clinical evaluation group. The statistical model used to evaluate these data is referred to as the ordinary least squares regression model. This model is not biased by the fact that the two groups, of daily and 48 hour supplement use, have different number of subjects (*n* = 100 vs. *n* = 30, respectively). If each group has a sufficient number of subjects to determine significance, the overall model will be sufficiently powerful in determining the significance between groups, thus minimising the possibility of statistical bias when comparing daily use of the supplement, with use every 48 hours.

The choice to take supplements daily, or on alternate days, can be driven by information derived from online content, by personal preferences, or by financial constraints. Regardless of a reason, there was to date no published evidence to support the impact of the dosing regimen on clinical efficacy and benefits when taking hydrolysed collagen supplements. We present here the first clinical evidence, measured against placebo, that proves that taking a supplement on alternate days will decrease the benefits and reduce efficacy for skin collagen, wrinkles, and skin tone. We have shown that taking the AC supplement daily is more efficacious than use every 48 hours, and this should be borne in mind by consumers who wish to vary their dosing regimen.

## 5. Conclusion

We have shown that an AC supplement, when consumed daily over 12 weeks, can improve collagen content in the dermis. Furthermore, there is an association between collagen fibres in the dermis and aging features such as hydration, elasticity, and wrinkles. We have extended this to include trichoscope and clinical assessment of scalp and hair condition. We have shown that the efficacy of the supplement is directly dependent on dose regimen, with subjects who take the supplement every 48 hours show less efficacy than subjects who take the AC supplement daily. These data clearly demonstrate that taking a marine-sourced, hydrolysed collagen plus vitamin C supplement daily can improve skin, scalp, and hair.

## Figures and Tables

**Table 1:** Summary for the skin biophysical and clinical assessments.

Timepoint	Placebo mean ± SD	*n*	*p* value change from baseline	AC treatment mean ±SD	*n*	*p* value change from baseline	*p* value treatment vs. placebo
Confocal fragmentation							
Week 0	5.66 ± 1.32	52		5.81 ± 1.0	48		
Week 6	5.62 ± 1.27	51	*p*=0.82	4.34 ± 1.19	47	*p* < 0.01	*p* < 0.01
Week 12	5.65 ± 1.21	48	*p*=0.98	3.22 ± 1.24	46	*p* < 0.01	*p* < 0.01
Ultrasound HEP pixel count							
Week 0	280.1 ± 380.2	52		303.5 ± 351.0	45		
Week 6	984.8 ± 712.8	49	*p* < 0.01	1135.4 ± 804.5	44	*p* < 0.01	*p*=0.46
Week 12	772.3 ± 545.9	51	*p* < 0.01	1005.0 ± 765.5	47	*p* < 0.01	*p*=0.06
Ultrasound LEP ratio							
Week 0	1.07 ± 0.21	52		0.95 ± 0.26	46		
Week 6	0.97 ± 0.19	49	*p* < 0.01	0.79 ± 0.19	44	*p* < 0.01	*p* < 0.01
Week 12	0.99 ± 0.22	51	*p*=0.05	0.86 ± 0.24	47	*p*=0.04	*p*=0.05
Corneometer hydration							
Week 0	52.9 ± 15.8	52		59.9 ± 13.7	48		
Week 6	51.1 ± 11.8	52	*p*=0.26	64.4 ± 10.9	48	*p* < 0.01	*p* < 0.01
Week 12	45.3 ± 11.0	52	*p* < 0.01	68.2 ± 12.5	48	*p* < 0.01	*p* < 0.01
Elasticity R2							
Week 0	0.668 ± 0.08	52		0.682 ± 0.10	48		
Week 6	0.683 ± 0.09	52	*p*=0.215	0.823 ± 0.09	48	*p* < 0.01	*p* < 0.01
Week 12	0.682 ± 0.09	52	*P*=0.254	0.837 ± 0.11	48	*p* < 0.01	*p* < 0.01
Profilometry Rz							
Week 0	139.0 ± 10.6	52		136.9 ± 11.0	48		
Week 6	140.7 ± 10.8	52	*p*=0.25	126.3 ± 9.9	48	*p* < 0.01	*p* < 0.01
Week 12	144.1 ± 11.1	52	*p* < 0.01	110.1 ± 8.4	48	*p* < 0.01	*p* < 0.01
Glogau Scale							
Week 0	2.56 ± 0.67	52		2.56 ± 0.65	48		
Week 6	2.56 ± 0.67	52	n.c	2.50 ± 0.62	48	*p*=0.08	*p*=0.07
Week 12	2.56 ± 0.69	52	n.c	2.34 ± 0.64	48	*p* < 0.01	*p* < 0.01
Skin even tone							
Week 0	5.46 ± 1.29	52		5.60 ± 1.12	48		
Week 6	5.37 ± 1.25	52	*p*=0.3	4.58 ± 1.09	48	*p* < 0.01	*p* < 0.01
Week 12	5.21 ± 1.26	52	*p* < 0.01	3.81 ± 1.08	48	*p* < 0.01	*p* < 0.01

Descriptive and probability statistics for the skin biophysical parameters and expert visual grading scales are presented. The table shows the mean and standard deviation for each measurement, with statistical significance for change from baseline, and for treatment vs placebo (at Week 6 and at Week 12). A threshold of *p* ≤ 0.05 was applied in all cases.

**Table 2:** Daily use of AC supplement vs. use every 48 hours.

	Timepoint	Use of AC supplement every 48 hours			Use of AC supplement daily			Use of AC daily vs. use every 48 hours
		Mean ± SD	*n*	Treatment % change	Mean ± SD	*n*	Treatment % change	
Collagen fragmentation	Week 0	6.23 ± 0.56	13		5.81 ± 1.0	48		
	Week 12	5.57 ± 0.83	14	−10.6	3.22 ± 1.24	46	−44.6	*p* < 0.01

Elasticity R2	Week 0	0.721 ± 0.14	14		0.682 ± 0.10	48		
	Week 12	0.855 ± 0.12	14	18.6	0.837 ± 0.11	48	22.7	*p*=0.68

Profilometry Rz	Week 0	140.2 ± 10.7	14		136.9 ± 11.0	48		
	Week 12	122.7 ± 10.3	14	−12.5	110.1 ± 8.4	48	−19.7	*p* < 0.01

Skin even tone	Week 0	5.57 ± 1.02	14		5.6 ± 1.12	48		
	Week 12	4.11 ± 0.96	14	−26.3	3.81 ± 1.08	48	−31.9	*p*=0.03

Descriptive and probability statistics are presented. The table shows the mean and standard deviation at baseline and week 12, and percent change from baseline for each measurement at week 12. Statistical significance of the comparison of supplement use daily vs. use every 48 hours is presented. A threshold of *p* ≤ 0.05 was applied in all cases.

## Data Availability

The data used to support the findings of this study are available from the corresponding author upon request.
